# Community Garden Initiatives Addressing Health and Well-Being Outcomes: A Systematic Review of Infodemiology Aspects, Outcomes, and Target Populations

**DOI:** 10.3390/ijerph18041943

**Published:** 2021-02-17

**Authors:** Anna Gregis, Chiara Ghisalberti, Savino Sciascia, Francesco Sottile, Cristiana Peano

**Affiliations:** 1 UNESCO Chair in Sustainable Development and Territory Management, University of Turin, 10100 Turin, Italy; anna.gregis@unito.it (A.G.); chiara.ghisalberti@unito.it (C.G.); cristiana.peano@unito.it (C.P.); 2 Center of Research of Immunopathology & Rare Diseases, Nephrology & Dialysis, S. Giovanni Bosco Hospital, Department of Clinical and Biological Sciences, University of Turin, 10100 Turin, Italy; 3 Department of Architecture (DARCH), University of Palermo, VialedelleScienze, Edificio 14, 90145 Palermo, Italy; francesco.sottile@unipa.it; 4 Department of Agricultural, Forest and Food Sciences (DISAFA), University of Torino, Largo Paolo Braccini 2, Grugliasco, 10095 Torino, Italy

**Keywords:** community gardens, health promotion, well-being, urban greenspace, public health

## Abstract

Previous research has suggested that activities such as community gardens could offer a wide range of health benefits. The aim of the article is to systematically review the available literature to analyse the magnitude of the phenomenon, the geographical distribution, and the main characteristics in terms of health outcomes and target populations. The search addresses the question whether the activity in community gardens improves health and well-being outcomes of individuals. From the total amount of 7226, 84 selected articles showed that:(1) up to 50% are published by U.S. universities or institutions; (2) up to 44% of the studies considered “community gardens” as the main activity of the research focus; (3) one-third of the studies included adults; (4) almost 25% of the studies used “general health” as the main outcome when investigating the benefits of community gardens; (5) the percentage of studies that achieved their outcomes was heterogeneous among the different health dimensions. In conclusion, while a certain degree of heterogeneity in the used definition and outcome still exist, community gardens may be a viable strategy for well-being promotion in terms of psychological, social, and physical health and may be considered as an innovative urban strategy to promote urban public health.

## 1. Introduction

With the world population rapidly increasing, it is estimated that up to 70% of people will live in urban spaces in the next threedecades [1]. This trend has enormous implications for both human health and environmental impacts. Hence, urban policies supporting the promotion of sustainable and healthy lifestyles are urgently needed [2].

Previous research has suggested that activities such as community gardens could offer a wide range of health benefits. Indeed, across Europe, there are around three million individual allotment gardens. These kinds of initiatives are now spreading not only in Europe but all around the world [3].

The phenomenon of urbanization has been associated with negative impacts on human health, such as mental illness and obesity [4]. In this regard, a growing body of research is suggesting that the availability of urban green space close to the home is associated with longevity and general improvement on health in the users [5]. Gardening might play a key role not only in preventive health, both in urban and suburban areas, but it is becoming an important policy strategy for “sustainable urban development” [6].

Sustainable urban development encompasses several urban settlement theoretical frameworks, including the concepts of “Sustainable City”, “Low-Carbon City”, “Healthy City”, “Livable City”, “Smart City”, and “Green City” [7].

In a recent of analysis, Hui-Ting Tang and Yuh-Ming Lee stated that sustainable urban development aims “to achieve maximum development with minimum resource consumption and environmental impact to ensure the well-being of both humans and the Earth” [8].

Although community gardens are becoming more and more prevalent, there is still a lack of consensus when referring to some definitions. The terminology of “community garden” is probably the most diffused and can be considered as an umbrella for all the other typologies. It is conceived as a green space where individuals grow vegetables and food in a common and collective way [9]. This last aspect of the management of food growing marks a difference from the allotment gardens, instead considered as “a parcel of land acquired by individuals and/or family via a lease or rent for personal usage” [10]. Gardens can be found in many different areas, from parks in cities to countryside areas. The aims of the gardens can also vary. For our analysis, we chose to apply the terminology of “community gardens”. However, significant differences from the chosen terminology will be commented on when present.

### What Is the Link between Human Health and Well-Being and Community Gardens?

More than half a century ago, the World Health Organization (WHO) defined health as “a state of complete physical, mental and social well-being and not merely the absence of disease or infirmity” [11]. Although this definition has been debated for being overly inclusive and hard to achieve [12], it has the merit to broaden the medical definition of health beyond the simple absence of disease. For the purpose of our analysis, we applied the following definitions for health and wellbeing. Health is a state of being, whereas well-being aims to integrate mental health (mind) and physical health (body) resulting in more holistic approaches to disease prevention and health promotion [13].

The ‘exposure-effect’ of urban gardening on health and well-being has been largely investigated [14,15,16,17,18]. Available studies have shown that the incidence of various chronic and non-communicable diseases, including depression and anxiety symptoms, diabetes, and obesity, is affected by the availability and size of green spaces [19].

Since community gardens are considered as an activity conducted in a green space, they might be part of a multi-component intervention that involves many activities: gardening and physical activity (PA), using and enjoying a green space, food production and consumption, social interaction. This multiple set of activities has been shown to enhance the local environment and the community, promote general health and well-being [20]. Hence, gardening delivers benefits across the physical, psychological, and social dimensions of health [21].

From a social point of view, several research studies have demonstrated that gardening improves social ties, enhances community capacity and knowledge [22]. By proving a variety of emotional and social processes, gardening positively affects people’s well-being [23]. Other studies demonstrate that gardening has a clear relationship between stress and anxiety reduction, with evident improvements in mood [24], self-esteem, and satisfaction [25,26]. These benefits are linked to the social, psychological, and emotional sphere of an individual practicing gardening.

To offer a reliable overview, many activities have assessed the impacts in terms of social and psychological benefits, including dietary patterns, mostly investigating fruit and vegetable (F&V) consumption and dietary behaviors [26,27,28,29,30]. Most of them are linked to school gardens or activities targeting children. An interesting study proved that gardeners and their children were more willing to eat foods if they picked themselves [23].

The possibility that gardening offers individuals the opportunity for physical activity needs to be emphasized [28]. Indeed, especially during spring and summer seasons, research highlights a significant increase of physical activity as a result of the multiple tasks required, such as picking, growing, and daily management works, potentially leading to a health benefit (e.g., in terms of metabolic and cardiovascular conditions) [28]. In line with this information, Wood et al. [29] have found that beneficial health effects related to gardening can be observed with less than 30 min of daily physical activity. A significant result is a special legislation proposed and approved recently by the Danish government, which gives allotment gardens a permanent status, thus enhancing healthy living policies [30].

While the above-mentioned aspects have been supporting a promising role in the implementation of community gardens, still a degree of heterogeneity exists when referring to the type of urban gardening, outcomes of health impact, and included populations [31,32]. Indeed, an analysis of qualitative and quantitative aspects of the impact of community gardens on human health has the potential to guide future policy strategies and areas of future development. However, a comprehensive analysis investigating the granularity of the current scenarios is still missing [33].

In this study we aimed to systematically review the available literature investigating the impact of the community garden on human health and well-being, analyzing the magnitude of the phenomenon, the geographical distribution, and the main characteristics in terms of health outcomes and target populations.

## 2. Research and Methods

A detailed literature review has been developed a priori to identify articles that reported findings on health outcomes in individuals exposed to community gardening activity to reply to the Population/Patient/Problem, Intervention, Comparison, Outcome (PICO) question: does the activity in community gardens improve health and well-being outcomes of individuals?

Considering the topic of the research, the team identified two databases for the search: Scopus and PubMed. Both qualitative and quantitative studies have been included. The search included the international literature, which is published in English, from 2010 to 2020.The search was obtained by the string “health” AND “garden”.

Inclusion criteria of the studies included: (a) individual exposed to community gardening activity (b) the presence of health outcomes among the effects of the exposure (c) original research.

For this review, the terminology community gardening was used through our search and possible alternative nomenclature screened, with the understanding of defining community gardening as all the activities that entail foods and vegetable cultivation by individuals, with communal or individual management. It includes all types of “community gardens” such as school gardens, specific policy programs, prison gardens, as well as individual initiatives such as household gardens. The selection included articles that were assessing health outcomes linked to “community gardens” also in case this activity was not the only one involved in the study. No differences in the inclusion have been done concerning the settlement of the community gardens (urban areas or rural). Finally, the activities included in the study address individuals of any age and belonging to any cases, even specific cases such as prisoners or cancer survivors. In terms of health outcomes, authors considered all types of assessment, self-rated evaluations by gardeners or assessed by researchers, which showed impacts related to health (e.g., cardiovascular outcomes, metabolic conditions, body mass index, diet patterns, psychological impact) and well-being (social factors).

Exclusion criteria included: (a) all studies not meeting the inclusion criteria were excluded from this review. (b) literature reviews or commentary articles. (c) studies that assessed only dietary intake (e.g., fruit and vegetable consumption). The approach used was to review a minimum of 10 percent of titles, abstracts, and full text results, to offer an overarching critique of the strengths and limitations of existing health and community garden studies.

We attempt an infodemiology approach—defined as a strategy to investigate the distribution and determinants of information in an electronic medium, specifically the Internet-when analyzing the distribution and determinants of information in available publications, with the ultimate aim to inform public health and public policy.

To quantify the potential benefit of the community garden on the different dimensions of health and well-being, a radar chart analysis was applied. Chi-Square test was used to analyze the discrete variable. Microsoft excel 2010 (Microsoft, Redmond, Washington, DC, USA) was used to draw the radar plot. *p*-value is considered to be significant if less than 0.05.

## 3. Results

**Figure 1:**
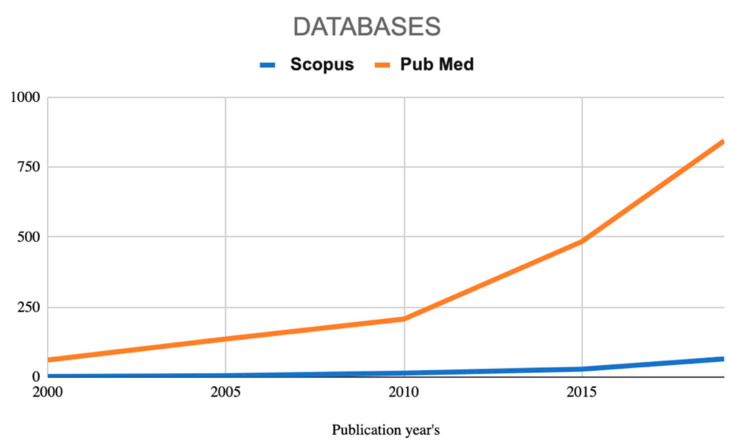
PubMed and Scopus publication trend between 2000 and August 2020.

The search obtained by the string “health” AND “garden”, identified 4800 articles on PubMed and 2426 on Scopus. Among these, the articles available in both databases were excluded. From the total amount of 7226, the present analysis focuses on the 84 selected articles meeting the inclusion criteria and pertinent for this analysis. As shown in Figure 1, there is a growing interest in the topic, as supported by the increasing number of published studies over the last 10 years in both Scopus and PubMed. However, the trend seems more evident when analyzing data from PubMed.

**Figure 2:**
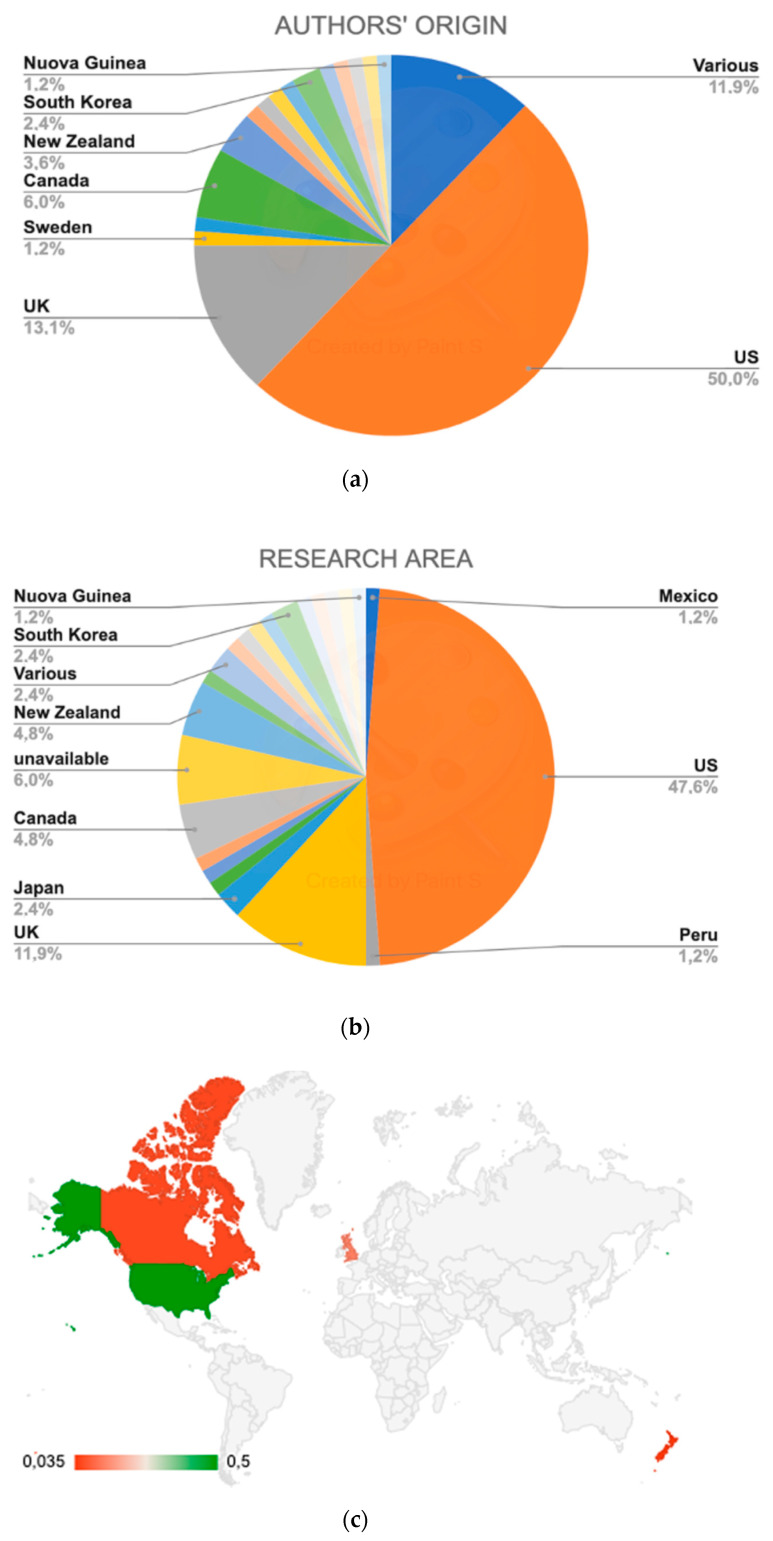
(**a**) Origin of the publications’ authors; (**b**): Geographical areas of research; (**c**): Geographical interest of the research topic.

Out of 84 analyzed studies, half (50%) are published by U.S. universities or institutions, followed by the United Kingdom (13,1%). Up to 12% of the published studies are the results of international collaborations. No significant difference was observed when comparing the location of researchers/institutions and the areas where the research was conducted (Figure 2).

**Figure 3:**
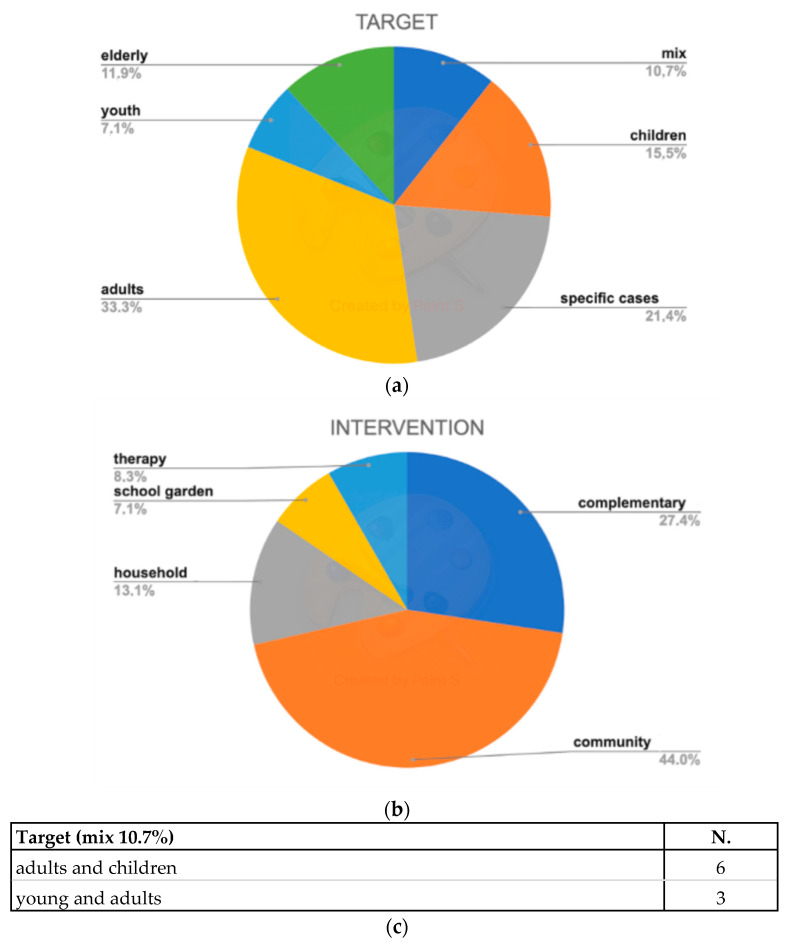
(**a**) Type of activity/intervention; (**b**)Garden-based activities target to different groups. (**c**) details on the mixed age-groups.

Participating in the activity of a community garden could represent the main focus of the research. Alternatively, being part of the activity of a community garden could be part of a composite intervention to improve health/well-being through other activities (e.g., change in diet, change in PA, etc.). In detail, we found that up to 44% of the studies considered “community gardens” as the main activity of the research focus (Figure 3a). Almost one-third of the research, instead, gardening was considered as part of multiple activities, (labeled as “complementary), to assess whether these practices might confer any health benefits. Up to 10% of the studies focused on household gardening. Finally, around 8% of the total studies focused on both horticultural therapy and school gardens.

When stratifying results for the studied population, results are shown in Figure 3b. One-third of the studies included adults; one-fifth is related to specific cases such as prisoners and individuals affected by different diseases, with cancer the most represented. Relevant interest is shown in children and youth and more than one on ten is addressed to the elderly. Finally, more than 10% of the studies investigated mixed groups, with 6 studies focusing on adults and children (most of the time mothers) and just 3 focus on youth and adults (Figure 3b,c).

### Health Outcomes

**Figure 4:**
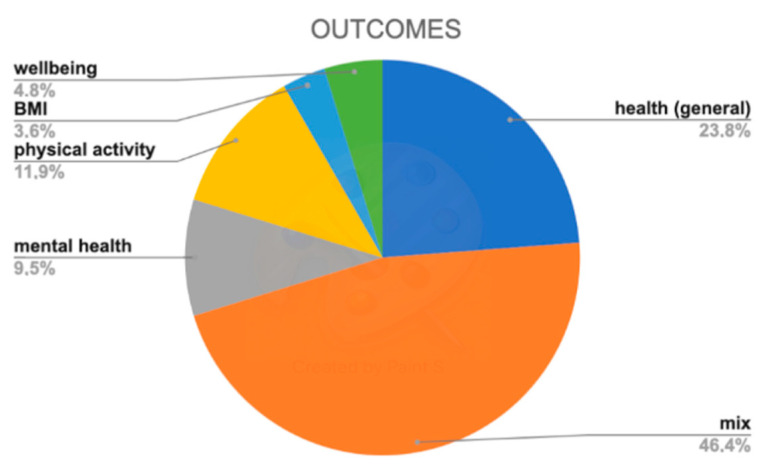
Different outcomes distribution grouped by main categories.

Figure 4 shows the distribution of different outcomes measured in each study.

Almost 25% of the studies used “general health” as the main outcome when investigating the benefits of community gardens. Around 10% reported benefits both in terms of mental health and physical activity. A marginal amount of the studies evaluated the impacts in terms of body mass index (BMI) and general “well-being”. It is worth noting that up to 46% of the studies used composite outcome definitions when evaluating the impact of community gardening on health and wellbeing. Table 1 provides the details on the different indicators selected for the assessment of the impacts generated by the garden-based activity in studies with composite outcome definition.

**Figure 5:**
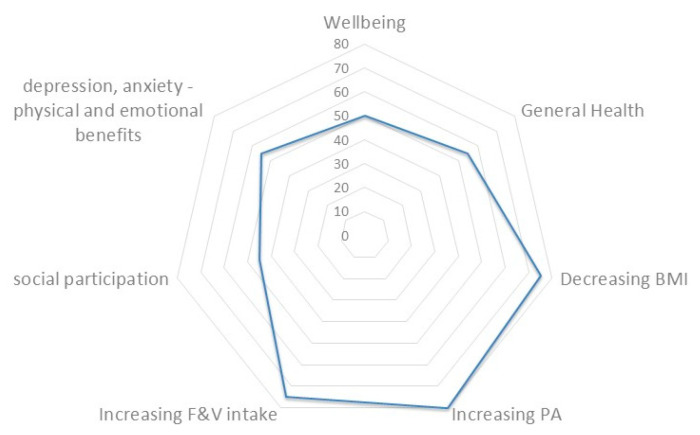
Spider chart providing a graphical representation of the percentage of studies meeting their outcomes subgrouping them for analyzed health dimensions.

When attempting to quantify the potential benefit of the community garden on the different dimensions of health and well-being, a radar chart analysis was performed (Figure 5).

The data length of each spike is proportional to the percentage of studies meeting their outcomes sub-grouping studies according to the used outcome. Studies with composite outcomes were computed separately taking into account the different results when provided. Figure 5 showed that the percentage of studies meeting their outcome was heterogeneous among the different health dimensions, with reducing the BMI and increasing physical activity and consumption of fruit and vegetables, the dimensions more often observed in studies achieving their outcomes when compared to the other groups (*p*<0.05).

## 4. Discussion

In line with the urban sustainable development concept, urban gardening is a key player of a trend towards more green areas in cities, consumption of organic, locally grown food, and a closer link with one’s own living environment [8].

Our analysis showed that there is an overall increased interest in the field of community gardening, especially in some areas such as U.S. and U.K. Interestingly, while several initiatives on community garden are well established in European cities [34], this interest did not seem to be paralleled with scientific publications. This aspect is worth mentioning as it can suggest some degree of mismatch between initiatives operating in real-life settings and academic activities. Similarly, while the U.S. and U.K. are both in the list of top countries by number of scientific and technical journal articles, China has displaced the U.S. as the world’s top research publisher in science and engineering [35]. However, this trend was not observed in our analysis. Besides, while it was out of the scope of our analysis to investigate the differences in retrievable studies on the topic when comparing Scopus and PubMed, one could speculate that PubMed might index journals in the field of medicine not necessarily included in Scopus.

While the associations between green space and human health have been summarized in several frameworks over the last years [36], we aimed to place these ‘exposure-effect’ relations in the broader context of urban sustainable development. In fact, despite the increasing interest of urban community gardens, planning guidelines concerning specific objectives and activities, target populations, and, more critically, outcomes applied to investigate potential benefit on health and well-being are still debated.

As expected, we found a marked level of heterogeneity in terms of those aspects. Indeed, also the terminology used to refer to community gardening present a marked level of variability, potentially impacting the extrapolatable potential of the results. Challenges have been encountered when trying to systematize the health and well-being outcomes and impacts of garden-based interventions computing together results from different studies. Studies coming from different disciplinary approaches offer inconsistent and different outcome measurements. For instance, studies investigating specific target groups such as indigenous people and tribes [31] aimed to assess health using wider and multi-dimensional indicators. Conversely, some other studies used focuses on disease-specific indicators that might be applicable only in some specific settings [32]. An integrated framework able to assess the different indicators in a harmonized way should be warranted in future research to assess the impact of community activities such as gardening taking into account different dimensions.

Our analysis highlights some criticism to be addressed before planning future research in the field.

First, the duration of the interventions varied widely across the studies. Nonetheless, by assessing the frequency and the duration of the garden-based intervention some authors suggested those variables did not significantly affect the potential benefit on participants health [29]. This observation is relevant as it might suggest that even a short-term intervention might lead to some health benefits, making community gardening activity more easily implementable into daily urban routines.

Secondly, the type of intervention and seasonality. It is evident that seasonality affects the overall development of community gardening and potential outcomes need to take this aspect into account [33].This is particularly relevant, for instance, when outcomes such as physical activity are analyzed.

Thirdly, the reported studies vary widely in terms of methodology. In the majority of the studies, a control group is not reported, creating limitations and introducing bias, so reducing the possibilities to detect possible differences. When available [25], control groups are represented by non-gardeners or individuals living in the same neighborhood as gardeners.

Additionally, a marked variability across studies is represented by heterogeneity in data collection, with many studies being based on self-reporting [33]. While this method can increase participation in terms of sample size, it might lack reproducibility.

Despite the already mentioned heterogeneity, some considerations are worth noting. Firstly, our analysis showed that reducing the BMI, increasing physical activity, and fruit and vegetables intake are the dimensions more often observed in studies achieving their outcomes when compared to the other groups. On the one hand, these observations might be not surprising, as those indicators might be more easily quantifiable when compared to other social or emotional dimensions. On the other hand, these findings might pave the way for future research providing the basis and rationale for future reproducible and scalable approaches.

One novelty of our analysis is based on the comparison of the target populations when focusing on age. Some studies [33] targeted different age groups, including the elderly. Some heterogeneity in terms of impacts on health, especially in terms of mental health, cognitive functioning, and physical health was found in studies in which a comparison of results across age groups was possible [37]. This concept is further supported in an analysis specifically designed to demonstrate the therapeutic effects of horticulture in older adults [38]. Thereby, the authors suggest that it is important to consider the multiple demographic variables which may affect the results of the research.

When focusing on the impact of community gardening on children, our analysis reported that the majority of the studies focused on the implementation of school gardens or specific garden-based initiative [39]. Some of the included studies considering gardening as a complementary activity part of wider programs also including food education. These studies [38,39,40] mainly focused on dietary intake, specifically fruit and vegetable consumption. It is interesting to notice that studies are consistent when analyzing outcomes related to different age groups: the major assessment in elderly population is focused on physical activity and mental health; conversely, in children, the evaluation is mainly based on dietary patterns.

## 5. Conclusions

The present study seems the first systematic attempt to assess whether community gardens provide health and well-being benefits. Despite the relevant degree of variability, our results are consistent in showing a growing interest in community gardening as a potential tool to improve health and well-being outcomes across different group ages, geographical areas, target populations, and indicators.

All in all, community gardens may be a viable strategy for health promotion in terms of physical, social, and psychological dimensions and it may be considered a complementary urban strategy to promote urban public health [41,42].

Promoting and enhancing a coherent, systematic, and interdisciplinary impact assessment approach in community gardens will help actors and main stakeholders to design future policy strategies. Besides, an increase in green space in urban spaces may foster environmental sustainability and strengthen neighborhoods and community capacity and capital [43,44]. This is particularly true in an urban setting, with rates of obesity, air pollution, and cardio-vascular diseases increasing rapidly in the most populated areas. The identification of local-based solutions contributing to the improvement of public health is therefore highly needed. Furthermore, involving citizens in an engaging activity may enforce processes of community building, with the result of strengthened social ties.

## Figures and Tables

**Table 1:** Details of the main outcomes shown in studies with composite outcome definition (46.4% of the total).

F&V intake and PA	BMI Secondary outcomes: child BMI, and adult hand strength, self-reported physical and mental health, diabetes control and food security.
Dietary intake, mental and physical health, sense of community and social support, long-term maintenance	Participant accrual, retention, and satisfaction rates. Secondary outcomes (i.e., vegetable consumption, physical activity, performance and function, anthropometrics, biomarkers, and health-related quality of life)
weight status, vegetable and sugar sweetened beverage consumption, PA and sedentary behavior	health and personal growth, mental well-being
mental health and wellbeing	F&V consumption, waist circumference
dietary intake, mental and physical health; sense of community and social support; long-term maintenance	health and emotional well-being
mood and general health	quality of life and emotional well being
dietary intake, PA, anthropometrics, blood values, and skin carotenoids	nutrition and BMI
health, well-being and PA	access to food, nutrition, PA and mental health
depression, anxiety—physical and emotional benefits	nutrition and PA
PA, height, and weight, dietary habits.	F&V intake, PA quality-of-life, and physical function
F&V intake, risk of obesity and diabetes	Diabetes
dietary intake, obesity, and metabolic disease risk	diet, PA, and quality of life
Children’s length/height-for-age z-scores, micronutrient status, dietary intake, dietary diversity and other indicators of child growth, development and morbidity	F&V consumption, obesity
BMI, waist circumference, blood pressure, hemoglobin A1c, vitamin D, low-density lipoprotein cholesterol, and household food security	Diabetes
chronic stress	dietary intake and obesity risk
psychological distress and social participation	emotional well-being, physical health.
mental health, cognitive functioning and physical health	cognitive ability
PA, mental health, and stress management; weight, diabetes	dietary intake, BMI
health and well-being	stress, physical functions, mental health
dietary behaviors, PA, mental health, and social relationships	food security and well-being
cognitive and nutrition	

PA, physical activities; F&V, fruit and vegetables; BMI, body mass index.

## Data Availability

Available upon request to the corresponding author.
